# Chemical Characterization, Evaluation of Antimicrobial Potential, and Cytotoxic Activity of *Thuja occidentalis* L. and *Myrtus communis* L. Essential Oils for Topical Applications

**DOI:** 10.3390/molecules31071225

**Published:** 2026-04-07

**Authors:** Elena Dănilă, Ioana Cristina Marinas, Madalina Diana Gaboreanu, Vlad Andrei Neacșu, Irina Titorencu, Minodora Maria Marin, Durmuş Alpaslan Kaya, Nafiz Çeliktaş, Mădălina Albu Kaya, Raluca Țuțuianu

**Affiliations:** 1 INCDTP—Leather and Footwear Research Institute, Collagen Department, 031215 Bucharest, Romania; elena.danila@icpi.ro; 2 The Research Institute of the University of Bucharest (ICUB), 050107 Bucharest, Romania; ioana-cristina.marinas@icub.unibuc.ro (I.C.M.); gaboreanu.diana-madalina@s.bio.unibuc.ro (M.D.G.); 3 Botany and Microbiology Department, Faculty of Biology, University of Bucharest, Splaiul Independentei 91-95, 050095 Bucharest, Romania; 4 Faculty of Chemical Engineering and Biotechnology, National University of Science and Technology Politehnica Bucharest, 1–7 Gh. Polizu Street, 011061 Bucharest, Romania; vlad_andrei.neacsu@upb.ro; 5 Institute of Cellular Biology and Pathology “Nicolae Simionescu”, 8 B. P. Hasdeu Street, District 5, 050568 Bucharest, Romania; irina.titorencu@icbp.ro (I.T.); raluca.tutuianu@icbp.ro (R.Ț.); 6 Advanced Polymer Materials Group, National University of Science and Technology Politehnica Bucharest, 011061 Bucharest, Romania; 7 Research Institute for Advanced Materials, Products, and Processes (CAMPUS), National University of Science and Technology Politehnica Bucharest, 060042 Bucharest, Romania; 8 Faculty of Agriculture, Hatay Mustafa Kemal University, 31060 Antakya, Turkey; dak1976@msn.com (D.A.K.); nafizcel@hotmail.com (N.Ç.)

**Keywords:** essential oils, *Thuja occidentalis* L., *Myrtus communis* L., antimicrobial activity, antibiofilm activity

## Abstract

In this study, we investigated the chemical composition, antimicrobial and antibiofilm activities, and cytotoxicity of *Thuja occidentalis* L. (thuja) and *Myrtus communis* L. (myrtle) essential oils (EOs) to evaluate their potential as natural alternatives for topical applications. Thuja EOs were extracted from fresh and dried leaves and cones, while myrtle EO was extracted from fresh leaves. GC-MS analysis revealed that myrtle EO was rich in oxygenated monoterpenes (58.2%), predominantly eucalyptol (41.86%) and α-pinene (20.98%). In contrast, thuja EOs were dominated by monoterpene hydrocarbons (55–82%), with α-pinene as the major component (29–66%), and their composition varied markedly with plant organ and moisture state. Myrtle EO displayed the most potent and broad-spectrum antimicrobial activity, with MIC values as low as 3.096 µL/mL against *Staphylococcus aureus* and *Escherichia coli*, and effectively inhibited microbial adherence. Thuja EOs showed selective activity, particularly against Gram-positive bacteria and *Candida parapsilosis*, with EOs extracted from fresh leaves and cones exhibiting the lowest MICs (1.95–7.8 µL/mL). All EOs did not show cytotoxicity to human keratinocytes at concentrations ≤125 µg/mL and, when diluted to 0.05%, indicated excellent skin compatibility in human patch tests. This study suggests that myrtle and thuja EOs, particularly myrtle, are promising and safe natural antimicrobial agents for dermatological and cosmetic formulations.

## 1. Introduction

Essential oils (EOs) are complex combinations of up to 300 different volatile organic compounds with various molecular weights, belonging to classes such as alcohols, phenols, aldehydes, ketones, esters, ethers or oxides, amines, amides, heterocycles and, most importantly, terpenes. The terpenes present in plants play an important role in resistance to diseases caused by fungi and bacteria [1]. Undoubtedly, new roles remain to be discovered for this large class of compounds, considering that only a small percentage of terpenes have been investigated to date. EOs are mainly used in the cosmetics and perfume industries due to their varied composition [2].

Understanding the chemical and biological characteristics of these extracts and their constituents is paramount in order to discover new and beneficial applications in human health, cosmetics, agriculture and the environment. EOs serve as substitutes for artificial antimicrobial agents, with their most important biological effects including antifungal, antiviral, antibacterial, and insecticidal effects, as well as chemoprotective effects against cancer and antioxidant capacities [3,4,5].

*Myrtus communis* L. (myrtle), a member of the Myrtaceae family, is an oil plant renowned for its essential oil, which has been used in traditional medicine for many centuries [6]. There are over 3000 different species in the Myrtaceae family, divided into 100 different genera. The *M. communis* shrub can reach a height of three meters, has aromatic evergreen leaves, and produces small black fruits along the stems and branches. Numerous studies have led to descriptions of the chemical makeup of myrtle in several countries, including Tunisia, Algeria, Albania, Italy, Iran, Greece, Egypt, Yemen and Turkey. The species is recognized for its variable chemical compositions in different countries [7]. Principal components most frequently reported in the literature for myrtle oil obtained from the leaves are 1,8-cineole (eucalyptol), limonene, linalool, α-pinene, β-caryophyllene, myrtenyl acetate, linalyl acetate and α-terpinolene [8,9,10]. Myrtle EO is currently used in pharmacology and the food and cosmetic industries, as well as in various medicinal and cosmetic applications due to its emollient qualities [11]. Previous studies report that myrtle EOs function as nematicides, insecticides, antibacterials and fungicides [12]. The antibacterial activity of myrtle extracts and EOs stems from their effect on the permeability of the bacterial cell wall and membrane, promoting the release of cellular contents outside the cell and leading to the disruption of essential membrane functions, including enzymatic activity [6]. A previous study showed that myrtle EO strongly inhibits the growth of Gram-positive bacteria, such as *Enterococcus faecalis*, *Staphylococcus aureus*, *Staphylococcus epidermidis* and *Mycobacterium smegmatis* [13].

*Thuja occidentalis* L. (thuja), part of the Cupressaceae family, is a plant native to North America, known for its medicinal properties and also used as an ornamental plant in many parts of the world [14]. *T. occidentalis* EO has been demonstrated to exhibit antioxidant, antiviral, antispasmodic, anti-inflammatory, antitumoral, antibacterial, antifungal, antidiabetic, hypolipidemic and atheroprotective, gastroprotective, antiviral, and immune-stimulatory activities [15]. The main constituents reported in the literature for thuja EO are α-pinene, sabinene, α-thujone, bornyl acetate, β-thujone and fenchone [16]. Thuja has been shown to have antibacterial properties against a significant number of bacteria, such as *Salmonella* sp., *Enterobacter cloacae*, *Staphylococcus aureus*, *Escherichia coli*, *Pseudomonas aeruginosa*, *Klebsiella pneumoniae*, *Shigella flexeneri*, *Candida albicans*, *Proteus vulgaris*, and *Entercoccus faecalis*.

Although numerous studies have described the chemical composition and antimicrobial properties of myrtle and thuja EOs, these investigations are generally fragmented, focusing either on isolated chemical profiling or specific biological assays. Comparative analyses integrating organ-specific variability (leaves vs. other plant organs), post-harvest processing effects (fresh vs. dried material), antimicrobial and antibiofilm efficacy, cytotoxicity on human epidermal cells, and dermatological compatibility within a unified experimental framework remain limited. To the best of our knowledge, a systematic evaluation correlating chemotype-dependent compositional differences with both microbiological performance and topical safety assessment for these two species has not been comprehensively addressed.

For evaluating the antimicrobial activity, six representative microorganism strains frequently involved in skin and soft tissue infections were selected. *S. aureus* is the most common bacterial agent worldwide in skin infections and is associated with a wide spectrum of clinical skin manifestations, ranging from impetigo to abscesses and furunculosis, including methicillin-resistant forms (MRSA) that complicate treatment [17]. Biofilm-associated infections are also caused by the commensal and opportunistic pathogen *S. epidermidis*, especially in wounds and on medical equipment [18]. Due to its inherent resistance to several antibiotics, *P. aeruginosa* is implicated in burn and chronic wound infections [19]. Despite being less skin-specific, *E. coli* can colonize infected wounds [20]. Yeast strains like *C. albicans* and *C. parapsilosis* are often found in skin infections and promote biofilm development on injured tissues [21]. According to clinical investigations, skin and soft tissue infections are common, with *S. aureus* being the most frequently isolated organism and *Candida* yeasts being involved in mixed infections [22].

Therefore, the present study adopts an integrated approach to bridge this gap and provide a more translational perspective for the rational development of EO-based topical antimicrobial formulations. Herein, we report the EO extraction of from myrtle and thuja by classical hydrodistillation. The chemical composition of these EOs was characterized using gas chromatography–mass spectrometry (GC-MS). We evaluate their antimicrobial and antibiofilm activity to assess their potential as natural alternatives to conventional antibiotics. Furthermore, considering the lack of cytotoxicity on human keratinocytes, the two EOs are proven to be suitable candidates for topical cutaneous applications as well as for cosmetic products as natural preservatives.

## 2. Results and Discussion

### 2.1. Extraction of the Essential Oils

Table 1 presents the extraction yield for thuja and myrtle EO.

Regarding thuja, a substantial increase in oil yield was observed as a result of drying process. Thus, drying is a good option for keeping therapeutic herbs over time. This study suggested that air drying at room temperature might be a better method for obtaining a high essential oil yield and α-Thujene and β-Thujene content.

The best extraction yield for thuja was obtained from dried cones (Table 1). Previous studies showed that, in the case of thuja EO, dried materials, including cones, offer a high concentration of EOs. The data presented for all EOs are similar to those reported in the literature [23].

### 2.2. Chromatographic Analysis

GC-MS analysis of the myrtle EO revealed 11 components, accounting for 97.92% of the total chromatographic area (Table 2, and Figure S1). The remaining 2.08% represented peaks with areas less than 1%, with a very high signal-to-noise ratio, which made identification impossible. The oil composition is dominated by monoterpenic constituents, amongst which oxygenated monoterpenes are predominant. These account for 58.2% of the total composition, with eucalyptol as the principal component (41.86% of the total area). The high eucalyptol content suggests the oil belongs to a cineole-rich chemotype, which is typical of several Myrtaceae species [24]. Other relevant oxygenated monoterpenes include α-terpineol (7.70%) and linalool (4.95%), while myrtenol and geraniol are present only in minor amounts (<2%). Oxygenated monoterpenes are frequently reported to exhibit stronger antimicrobial activity than hydrocarbon monoterpenes due to their higher polarity and enhanced interaction with microbial cell membranes [25].

Monoterpene hydrocarbons represent 22.55% of the oil, with α-pinene as the dominant hydrocarbon fraction (20.98%). *p*-Cymene is present only in a minor proportion (1.57%). The significant proportion of α-pinene may also contribute to its biological activity, as α-pinene has demonstrated antibacterial effects against both Gram-positive and Gram-negative bacteria, mainly through membrane disruption mechanisms [26]. Finally, monoterpenoid esters constitute 17.17% of the total composition and are primarily represented by α-terpinyl acetate (6.99%) and myrtenyl acetate (4.72%), followed by geranyl acetate (3.03%) and linalyl acetate (2.43%). The presence of these esters may contribute indirectly to bioactivity, either through intrinsic antimicrobial properties or via in situ hydrolysis to their corresponding active alcohols. Overall, the chromatographic profile indicates that myrtle EO is strongly enriched in oxygenated monoterpenes, particularly eucalyptol, accompanied by significant amounts of α-pinene and monoterpenoid acetates. The predominance of oxygenated monoterpenes, particularly eucalyptol, suggests that the antimicrobial activity observed for myrtle EO is likely associated with membrane permeability alteration, leakage of intracellular contents, and the disruption of microbial metabolic processes mechanisms commonly reported for terpene-rich essential oils.

In the case of thuja EOs, GC-MS analysis allowed for the identification of 94.55–100% of the total composition across the four samples (Table 3, and Figures S2–S5). The compositions of all thuja EOs are strongly dominated by monoterpenes, although marked quantitative differences are observed depending on both plant part (leaves vs. cones) and moisture state (fresh vs. dry material). Monoterpene hydrocarbons represent the principal fraction in all samples, ranging from 55.48% (fresh cones) to 81.95% (fresh leaves). The predominant compound in all samples is α-pinene, accounting for at least 29% of the composition in all samples and reaching 65.78% in the EO extracted from fresh leaves. The predominance of α-pinene is consistent with the literature reports on thuja species and other Cupressaceae [27,28,29]. The EO extracted from fresh leaves exhibited the highest proportion of monoterpene hydrocarbons (81.95%), whereas that from fresh cones contained substantially lower amounts (55.48%) but higher proportions of sesquiterpenes (19.04). This difference likely reflects organ-specific terpene biosynthesis, as the expression of terpene synthase enzymes varies with the tissue types in conifers [30]. It is important to note that drying reverses this trend by increasing the total monoterpene hydrocarbons content in the EOs extracted from cones (from 55.48% to 72.98%), mainly due to increases in α-thujene, β-thujene, and γ-terpinene contents, while reducing the relative abundance of sesquiterpenes. Such shifts are commonly attributed to post-harvest biochemical transformations and differential volatility, as monoterpenes may be either lost or concentrated depending on the drying process employed [31,32].

Oxygenated monoterpenes are present at low levels in the EOs extracted from fresh material (1.51–1.54%) but increase considerably in the dried cones EO (11.52%). The most notable increase is seen for terpinen-4-ol (from 1.18% in fresh cones EO to 10.68% in dried cones EO). This increase may be explained by the oxidative conversion of precursor hydrocarbons, a phenomenon frequently reported during the post-harvest processing of aromatic plants [33]. Oxidation and rearrangement reactions during air-drying can lead to enrichment in oxygenated derivatives, which are generally more stable and less volatile than their hydrocarbon precursors [34]. The relatively low content of oxygenated monoterpenes in the EO extracted from leaves suggests either that monoterpene oxidation is less pronounced in non-reproductive tissues or that leaves preferentially accumulate hydrocarbon monoterpenes. Monoterpenoid esters (1.75–4.9%) are minor constituents in all samples, with α-terpinyl acetate and borneol acetate being consistently detected. The slightly higher ester content in fresh cones EO (4.9%) may reflect organ-specific terpene metabolism associated with reproductive structures. In conifers, reproductive tissues often accumulate volatile terpenes involved in ecological interactions, such as defense against herbivores and pathogens or the modulation of microenvironmental interactions [2]. The esterification of terpene alcohols is enzymatically regulated and can serve for the modulation, storage, or detoxification of reactive alcohol intermediates [35]. The moderate decrease upon drying could be due to partial hydrolysis or volatilization.

Sesquiterpene hydrocarbons are particularly abundant in dry leaves EO (17.77%) and fresh cones EO (19.04%), with caryophyllene, *cis*-thujopsene, and humulene as major representatives. Oxygenated sesquiterpenes show pronounced variability, particularly cedrol, which varies between 5.40% (fresh leaves EO) and 16.52% (dried leaves EO). The increase in oxygenated sesquiterpenes in dried leaves EO (19.11%) may reflect oxidative processes similar to those affecting monoterpenes. Sesquiterpene alcohols are less volatile and therefore tend to become relatively enriched when lighter monoterpenes evaporate during drying [35].

Overall, all four EOs can be described as α-pinene-dominant chemotypes, with substantial contributions from cedrol and thujene derivatives. However, the quantitative profile is heavily influenced by the plant part used for extraction, as well as whether the plant material was dried or not before processing. Fresh leaves result in a strongly monoterpene hydrocarbon-rich EO, with α-pinene content reaching up to 65.78%; EO from dried cones shows an increase in oxygenated monoterpenes, notably terpinen-4-ol, while EO from dried leaves shows an increase in oxygenated sesquiterpenes, with a cedrol content of up to 16.52%.

The components identified by GC-MS for both EOs are similar to previous reports, demonstrating the high purity of the oils obtained [13,36].

### 2.3. Antimicrobial Activity

The antibacterial activity of the investigated EOs correlates strongly with their chemical profile, specifically the presence and percentage of important terpene compounds known for their biological properties. Myrtle EO was the most active, particularly against *S. aureus*, *E. coli*, and *Candida* sp., demonstrating broad-spectrum antibacterial action according to the qualitative assay. Thuja EOs isolated from fresh leaves were the most effective against *Staphylococcus* sp. and *C. albicans*, while their effectiveness was significantly reduced against the tested Gram-negative pathogens Myrtle, and thuja EOs obtained from fresh cones were the most efficient against Gram-negative bacteria, particularly against the *E. coli* strain (Table 4).

**Figure 1:**
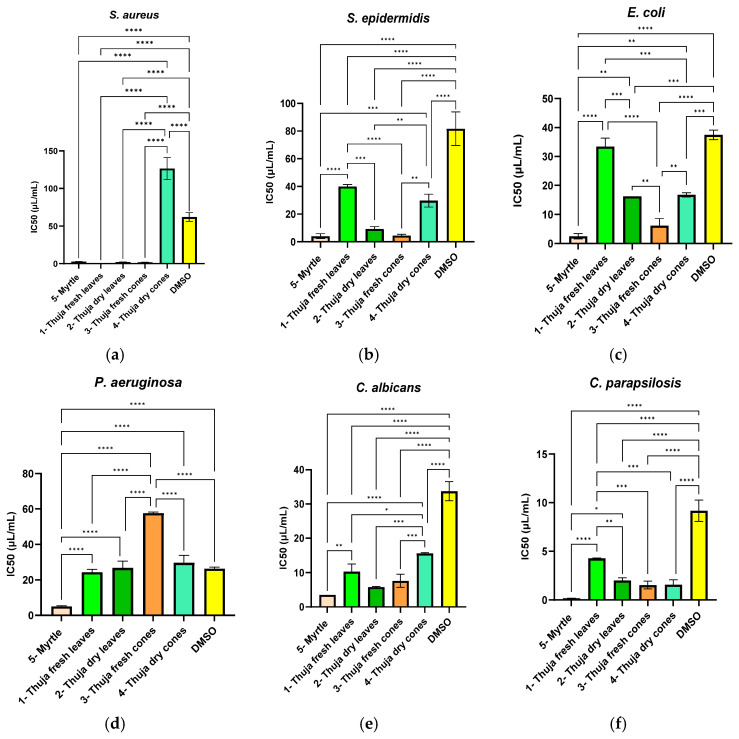
Antimicrobial activity was expressed in IC_50_ values, and the correlation with the activity of the solvent used is highlighted against (**a**) *S. aureus*, (**b**) *S. epidermidis*, (**c**) *E. coli*, (**d**) *P. aeruginosa*, (**e**) *C. albicans* and (**f**) *C. parapsilosis* strains. Statistical analysis of the data was performed using one-way ANOVA, followed by Tukey’s test for multiple comparisons. Data presented represent the mean of three independent experiments (*n* = 3), and the error bars illustrate the standard deviation of the means. Significant differences were indicated as * *p* < 0.05, ** *p* < 0.01, *** *p* < 0.001 and **** *p* < 0.0001.

Figure 1 shows the IC_50_ values (µL/mL) of the formulations evaluated against six reference microorganisms: Gram-positive bacteria (*S. aureus*, *S. epidermidis*), Gram-negative bacteria (*E. coli*, *P. aeruginosa*), and yeasts (*C. albicans*, *C. parapsilosis*). In general, myrtle EO had the strongest antibacterial activity, with the lowest IC_50_ values for tested all pathogens.

The MIC values demonstrated the EOs’ ability to inhibit bacterial growth. Table 4 illustrates the EOs’ inherent antibacterial activity by displaying MIC values that are lower than those for the control solvent (DMSO). Myrtle EO had the lowest MIC values for the majority of the tested isolates, demonstrating greater inhibitory efficacy than thuja EOs. EOs extracted from fresh thuja leaves and cones showed moderate activity, while EOs isolated from dried cones exhibited the highest MIC values but were still lower than the solvent.

The MMC parameter reflects the ability of EOs to produce a microbicidal effect. The values highlighted in green correspond to MMCs lower than the solvent, indicating a true lethal impact on microorganisms (Table 4). For this parameter, myrtle EO stood out with the lowest MIC values, suggesting superior microbicidal efficacy. Thuja EOs presented selective microbicidal behavior, particularly against *Staphylococcus* sp. strains., and *Candida* sp. was less effective than myrtle, particularly the samples from dry plant material.

The GC-MS profile revealed that α-pinene was the most abundant compound in both fresh (29.12–65.78%) and dried (30.02–33.65%) raw material, followed by cedrol, β-thujene, caryophyllene, 3-carene, cis-thujopsene and terpinen-4-ol.

The presence of sesquiterpenes like caryophyllene may contribute to a synergistic effect, which explains why *S. aureus* and *Candida* spp. have relatively low MIC and MMC values [37]. The EO extracted from fresh thuja leaves was the most effective, with MIC values ranging from 1.95 to 125 µL/mL, being notably efficient against Gram-positive bacteria. The EOs extracted from dry cones were shown to be the most efficient against yeast strains due to their high content of oxygenated monoterpenes (approximately 11.52%). The modest antimicrobial activity of thuja leaves EO in comparison to the cones suggests that oxygenated monoterpenes may be responsible for its antibacterial properties. Based on the very limited data found in the literature, it was observed that the EO of *T. occidentalis* had an antifungal effect against *C. albicans*, *C. tropicalis*, *C. glabrata*, *Curvularia* spp., *Exserohilum* spp., *A. flavus*, *A. niger*, and *Bipolaris* spp. [23,38], as well as antibacterial activity against *L. monocytogenes*, *B. cereus*, *S. aureus*, *S. epidermidis*, *P. aeruginosa*, *E. cloacae*, *K. pneumoniae*, and *E. coli* [23,39]. After converting the MIC values from µL/mL to mg/mL based on the density of EOs, the results of the present study for thuja fresh leaves were in accordance with the literature data [23], which report antibacterial activity in the range of 1.1–2.7 mg/mL. The highest concentrations of components in the myrtle EO were assigned to eucalyptol (41.86%) and α-pinene (20.98%), followed by α-terpineol, α-terpinyl acetate, linalool, and myrtenyl acetate. This composition explains the pronounced antimicrobial activity observed against all tested strains, including *E. coli* and *P. aeruginosa*. Eucalyptol is recognized for its broad-spectrum antimicrobial activity, acting by increasing the cell membrane permeability and inducing the loss of cytoplasmic constituents [40]. α-Pinene and linalool additionally contribute through similar mechanisms, and the synergistic interactions between these compounds can explain the low MIC and MBC values obtained for myrtle EO compared to thuja EOs [41].

The literature indicates a strong antibacterial profile for *M. communis* EO, with moderate antimicrobial activity that varies depending on the tested microorganism and the geographical origin of the collected plant. EO obtained from two areas in Palestine demonstrated different MIC values for most of the strains studied (MRSA, *S. aureus*, *K. pneumoniae*, *E. coli*, *P. vulgaris*, *P. aeruginosa* and *C. albicans*). *P. aeruginosa* was the most resistant species, while the antifungal activity against *C. albicans* was comparable for both EOs [42]. Other research has found that this essential oil has antifungal properties against *P. digitatum* and *A. niger* [43], as well as antibacterial properties against *S. aureus*, *P. aeruginosa*, *E. coli*, *P. carotovorum*, *L. monocytogenes* [44].

Among the tested thuja EOs, the one extracted from fresh leaves was distinguished by significant antimicrobial activity in the case of *S. aureus* (Figure 1a) and *S. epidermidis* (Figure 1b). These EOs had an efficiency comparable to myrtle EO, with no significant difference (*p* > 0.05). The lowest IC_50_ values for the *S. aureus* strain were recorded for thuja fresh leaves, thuja dried leaves, and thuja fresh cones, indicating high antibacterial activity. In the case of the *S. epidermidis* strain, a different behavior was observed compared to *S. aureus*. The best antibacterial activity (minimum IC_50_) was highlighted for fresh thuja cones and myrtle, with both being significantly more effective compared to the DMSO control (*p* < 0.05). In contrast, the fresh thuja leaves EO had moderate efficiency, close to that of the solvent control (*p* > 0.05). The IC_50_ values obtained for *E. coli* revealed strong antibacterial activity for most of the tested samples (Figure 1c). The lowest IC_50_ value was observed for the myrtle EO, followed by the fresh thuja cones, indicating effective inhibition of bacterial growth. In the case of the *P. aeruginosa* strain, a lower antimicrobial activity was observed (Figure 1d). Myrtle EO presented the best antibacterial activity, followed by fresh thuja leaves EO, but only the IC_50_ value for sample 5 (myrtle) was significantly lower than that of the solvent used (*p* < 0.05). The IC_50_ values determined for *C. albicans* showed pronounced antifungal activity for most of the tested samples (Figure 1e). The lowest IC_50_ value was observed for myrtle EO. The EO extracted from dried thuja leaves and fresh thuja cones also showed significant antifungal activity compared to the solvent (*p* < 0.05). In contrast, dried thuja cones and fresh thuja leaves had higher IC_50_ values, indicating lower antifungal activity, though they were still significantly different from the solvent (*p* < 0.05). The highest IC_50_ value was recorded for DMSO, used as a solvent control, which confirms the lack of its antifungal effect. In the case of the fungal strain *C. parapsilosis*, the IC_50_ values were considerably lower than those observed for Gram-negative bacteria, suggesting a higher sensitivity to the tested treatments (Figure 1f). The lowest IC_50_ values were recorded for myrtle and thuja cones (both fresh and dry), which showed significant antifungal activity (*p* < 0.05) compared to the DMSO control. Additionally, dried thuja leaves showed high efficiency, close to that of the previously mentioned samples.

These results highlight the antimicrobial potential of myrtle EO as a natural broad-spectrum antimicrobial agent, while some thuja EOs may provide selective activity, especially against yeasts and Gram-positive bacteria. Tsiri et al. [45] highlighted the same antimicrobial potential, which was specifically selective against Gram-positive bacteria (*S. aureus*, *S. epidermidis*) and yeasts (*C. albicans*, *C. tropicalis*, *C. glabrata*).

The EOs’ influence on microbial adherence, which represents the initial stage of microbial biofilm development [46], was also evaluated (Table 4). EOs that exhibit MBEC values lower than both their MIC values and the MBEC value for DMSO are considered effective in inhibiting microbial adherence (values highlighted in blue in Table 4).

In general, all tested samples, especially myrtle EO and fresh thuja cones exhibited the lowest MICMA values on five out of six strains tested, ranging between 3.096 and 62.5 μg/mL, indicating high efficacy in inhibiting biofilms formed by *S. aureus*, *E. coli*, and *C. parapsilosis*. The MICMA values written in red (lower than the corresponding MIC values) indicate cases in which the concentrations required to inhibit biofilm formation were even lower than those required to inhibit planktonic cell growth. This suggests increased sensitivity of the biofilm formation process to the tested compounds or possible mechanisms of action that affect adhesion formation before inhibiting bacterial cell viability [47]. Notable examples include the effect of fresh thuja leaves EO on *S. epidermidis*, *E. coli*, and *P. aeruginosa*, as well as the effect of fresh thuja cones EO on *E. coli*, where MICMA values were significantly reduced. In the case of fungal strains (*C. albicans* and *C. parapsilosis*), only Sample 5—myrtle—showed MICMA values noticeably lower than DMSO, indicating moderate antibiofilm activity compared to the antibacterial effect.

The results suggest that EOs rich in oxygenated monoterpenes exhibit superior antimicrobial activity compared to those dominated by sesquiterpene and monoterpene hydrocarbons. Furthermore, the antimicrobial effects cannot be attributed solely to the major compounds, but rather to the synergistic interactions between the EO constituents. As highlighted in a study conducted by Miladinović et al. [48], the minor components of EOs play an essential role in antibacterial activity, even though they are only present in small quantities.

### 2.4. In Vitro Cytotoxicity Assessment: XTT and LDH Assays on Human Keratinocytes

**Figure 2:**
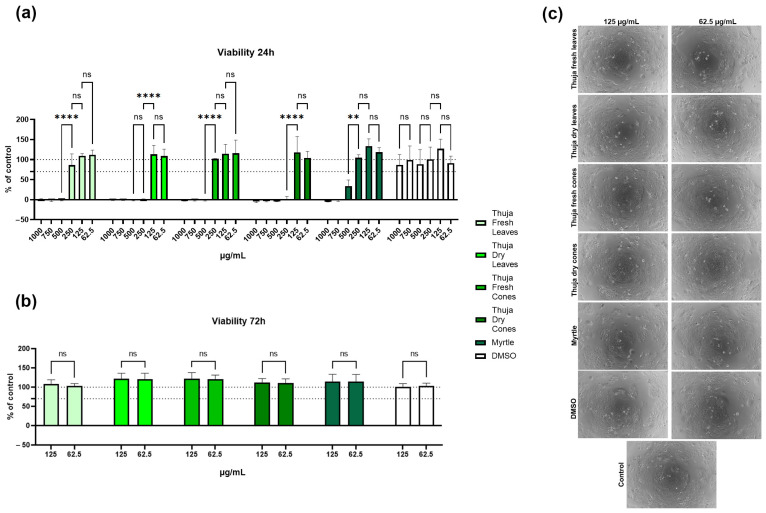
Human keratinocyte (HaCaT) viability after (**a**) 24 h and (**b**) 72 h of incubation with EO concentrations ranging between 1000 and 62.5 µg/mL, evaluated using XTT and expressed as percentage of control (untreated cells). The dashed line at 70% represents the threshold for cell toxicity. (**c**) Phase-contrast microscopy images of the cells after 3 days of cultivation under the corresponding experimental conditions, at 10× magnification. Data are presented as mean ± SEM (Standard Error of the Mean) (n ≥ 3), ** *p* < 0.05, **** *p* < 0.001, ns: not significant.

The viability of human keratinocytes from the HaCaT cell line was severely inhibited at the first three highest concentrations tested (1000, 750, and 500 μg/mL, respectively) for all five essential oils after the initial 24 h of incubation, as seen in Figure 2a. Furthermore, this effect was not caused by the presence of the vehicle (DMSO). The thuja fresh leaves, thuja fresh cones, and myrtle EOs showed a viability above 70% of the control at concentrations of 250, 125, and 62.5 μg/mL, and the values were significantly increased compared to the higher concentrations. For the other two samples (thuja dry leaves and thuja dry cones), keratinocyte viability was maintained at or above 100% of the control.

Considering the results regarding the antimicrobial activity of these EOs, for a prolonged effect on cell viability at 72 h, we only tested the non-cytotoxic concentrations: 125 and 62.5 µg/mL for each sample, together with the vehicle control. All EO samples supported cell proliferation at a similar level for the two dilutions (Figure 2b). These data were also supported by the phase-contrast images of the keratinocyte culture after 3 days of cultivation in the presence of the tested essential oils (Figure 2c).

**Figure 3:**
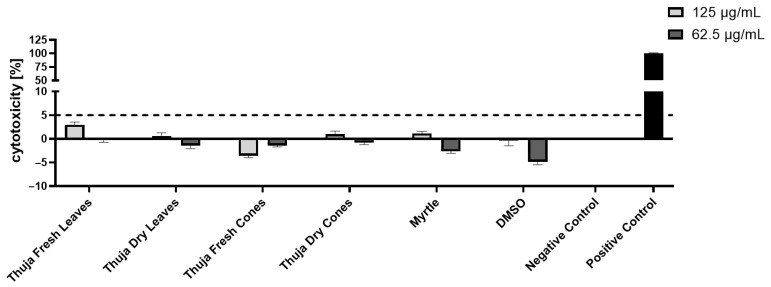
Cytotoxicity evaluation of EOs on human keratinocytes, using the LDH test. Data are presented as mean ± SD (standard deviation) (n ≥ 3). The dashed line at 5% represents the low-level threshold for lack of cytotoxicity.

In order to confirm the lack of a toxic effect for the two biologically active dilutions 125 and 62.5 µg/mL, we also checked the release of LDH in the culture medium. As seen in Figure 3, there was no significant effect observed on the human keratinocytes upon incubation with the selected concentrations of the five essential oils tested, with all cytotoxicity values being under 5%.

The viability assessment on human keratinocytes after 24 h exposure revealed that for the fresh leaves and cones of thuja EOs, as well as myrtle, the non-cytotoxic effect started at a higher concentration 250 µg/mL versus 125 µg/mL for the dried preparations. These results probably reflect the differences in their chemical composition, since recently, it has been reported that essential oils rich in sesquiterpenes and phenols were associated with a higher cytotoxicity on HaCaT, at 200 µg/mL [49].

### 2.5. Skin Compatibility Assessment by In Vivo Testing

Across all subjects, none of the tested thuja and myrtle EOs, nor the negative control, induced any clinically relevant signs of irritation or other adverse dermatological reactions at any observation time point. As expected, the positive control elicited the anticipated irritation responses, consistent with established diagnostic patch-testing guidelines [19].

Under the defined experimental conditions, the mean irritation values of all tested EOs were not significantly different from the negative control, but they presented significantly different values from the positive control. These irritation values were consistently below widely accepted thresholds indicative of dermal reactivity, supporting their classification as non-irritating. Similar outcomes have been documented in standardized 24 h occlusive human patch-test studies, in which test materials producing irritation scores in the minimal or zero range (e.g., scores near 0.0 on erythema/edema grading scales) are considered non-irritating and safe for topical application in human subjects [50]. Overall, the results of the present study demonstrate that, under the specified experimental conditions, the evaluated EOs (diluted to 0.05% in ethanol) exhibit very good skin compatibility, supporting their suitability for further applications. No significant differences in skin compatibility were observed among the thuja and myrtle EOs.

## 3. Materials and Methods

### 3.1. Material Sampling

In August 2025, thuja branches were collected from Romania and taken to the laboratory, where the leaves and cones were detached from the branches and stored at room temperature until extraction, which took place immediately (for fresh samples) and within 30 days of harvest (for dried samples). The extraction of thuja EO was carried out from both cones and leaves, fresh and dried. The fresh plant material was chopped into smaller pieces before extraction. Thuja (*Thuja occidentalis*) was collected by Elena Dănilă from the location 44°24′49.2″ N 26°7′13.6″ E. Their identity was confirmed by Carmen-Comanescu Pentronela and voucher specimens have been deposited at the herbarium of the Botanical Garden Dimitrie Brandza of the University of Bucharest (No. 412728).

Fresh myrtle leaves were collected from Turkey during the optimal harvest period, which coincides with the full blooming stage. The extraction was performed from fresh leaves only. Myrtle (*Myrtus communis* L.) was collected by Prof. Dr. Durmus Alpaslan Kaya from the location 36°10′39.3″ N 35°59′06.2″ E and is deposited in the HMKU Herbarium with the collector number A. Kaya-015. The accuracy of the identification was confirmed by Prof. Dr. Yelda Güzel.

### 3.2. EOs Extraction

**Figure 4:**
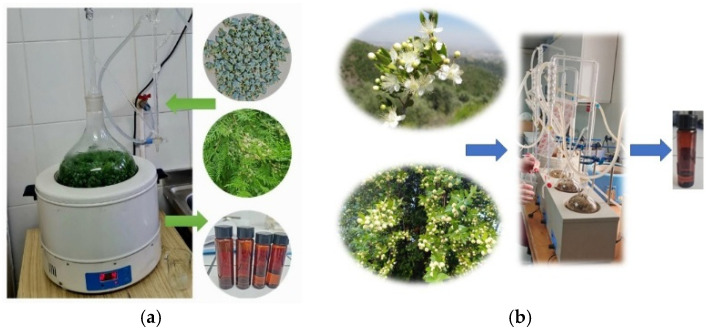
Extraction of (**a**) thuja EO (from leaves and cones) and (**b**) myrtle (from leaves).

The plant material was placed in a round-bottom flask to which a specific volume of distilled water was added (Table 1); the mixture was extracted for approximately 2 h using a Neo-Clevenger type apparatus (Adrian Sistem Lab SRL, Bucharest, Romania), during which the oil was collected in the oil trap. The apparatus was then left to stand for about half an hour to allow the oil to reach room temperature. Subsequently, anhydrous sodium sulfate was added to the collected oil to remove any residual water. The obtained samples were stored in amber vials in a refrigerator at 4 °C until analysis. The general extraction process for essential oils is illustrated in Figure 4.

### 3.3. Characterization of EOs

#### 3.3.1. Chromatographic Analysis

Thuja and myrtle EOs were analyzed using an Agilent System 8890GC gas chromatograph, (Agilent, Technologies, Wilmington, Germany) equipped with a GC/MSD 5977C mass spectrometer, and an HP-5MS inert column (30 m × 0.25 mm ID × 0.25 μm film thickness). The carrier gas used was helium with a flow rate of 1 mL/min and the following temperature program was used: 50 °C, with a gradual increase of 3 °C/min up to 220 °C. The mass spectra were analyzed using the. NIST MS Search v 3.0 program.

For compound identification, only peaks with a relative area ≥ 1% were systematically considered in order to ensure reliable assignments based on adequate signal-to-noise ratios and spectral matching confidence. Peaks below this threshold were examined but excluded from reporting when identification probabilities were low or chromatographic resolution was insufficient to avoid potential misassignments. In the case of thuja EOs, peaks over the 1% threshold in at least one sample were further cross-compared across all samples and subsequently included in the analysis even if their relative abundance was under 1% in other samples.

#### 3.3.2. Antimicrobial Activity

*Microbial Strains.* The reference microbial strains (*S. aureus* ATCC 25923, *S. epidermidis* ATCC 12228, *P. aeruginosa* ATCC 27853, *E. coli* ATCC 25922, *C. albicans* ATCC 10231, *C. parapsilosis* ATCC 22019) used in this study were purchased from authorized suppliers and are part of the strain collection of the Faculty of Biology, University of Bucharest.

*Qualitative Evaluation of Antimicrobial Activities*: The antimicrobial capacity was assessed using microbial suspensions that were prepared and standardized to 1.5 × 10^8^ CFU/mL, which is equivalent to the McFarland 0.5 nephelometric standard. These suspensions were generated from cultures grown on solid media for 18–24 h (Sabouraud dextrose agar for yeasts, Mueller–Hinton agar for bacteria). According to the Clinical and Laboratory Standards Institute (CLSI), a diffusimetric method modified for a 1:1 EOs stock solution in DMSO was used to assess the antibacterial activity of EOs. For each microbial strain examined, a solvent control (DMSO) was used. The agar medium inoculated with the tested strain was spotted with 5 μL of each EOs stock solution. The plates were incubated at 37 °C for 24 h. The diameter of the inhibition zone (DIZ) around the spot was measured, and the results were expressed as mean ± standard deviation for each EO tested in duplicate.

*Quantitative Evaluation of Antimicrobial Activity*: Serial binary microdilutions in liquid media (Sabouraud Broth for yeasts, Tryptone Soy Broth for bacteria) on 96-well plates were used for quantitative analysis. Each sample had a concentration range of 125–0.244 µL/mL, and stock solutions of EO were prepared 1:1 (*v*:*v*) in DMSO. The same working conditions were used for the solvent control (DMSO). Each well was inoculated with 10 µL of microbial suspension prepared from 18 to 24 h cultures, adjusted to a density of 1.5 × 10^8^ CFU/mL for bacteria and 1.5 × 10^6^ CFU/mL for yeasts. The MIC (minimum inhibitory concentration) values were determined by measuring the absorbance at 620 nm and macroscopically evaluating the growth and multiplication of microorganisms after 24 h at 37 °C. The data obtained were analyzed using the Inhibitor vs. Response–Variable Slope (four parameters) analysis function with Prism GraphPad 10.0 software, in order to calculate the IC_50_ (the concentration of the sample that inhibits the growth of 50% of the untreated microbial inoculum).

*Microbicidal Activity Assessment*: To measure the minimum microbicidal concentrations (MMC), 5 µL of culture was removed from each well and spotted on solid medium. The plates were incubated at 37 °C for 24 h. The MMC was defined as the lowest concentration at which no microbial growth was detected.

*Microbial Adhesion*: Microbial adherence was evaluated following methanol fixation and crystal violet staining (0.1%), which was performed after the quantitative investigation of antimicrobial activity. The absorbance at 490 nm of the adherent biomass that was stained and reconstituted in 33% acetic acid was measured.

*Statistical Analysis*: Data were expressed as means ± standard deviation (SD) from duplicate or triplicate analyses. GraphPad Prism 10.0 was used for statistical analysis. Brown–Forsythe and Welch ANOVA, followed by Dunnett’s T3 test for multiple comparisons, were used to compare the IC_50_ values of EOs and the solvent. A significance threshold of *p* < 0.05 was established. Statistical significance was considered at *p* < 0.05.

#### 3.3.3. Cell Culture Techniques

Human keratinocytes from the HaCaT cell line (CLS, Eppelheim, Germany) were cultured according to the manufacturer’s instructions. Briefly, the cells were cultivated in high glucose DMEM (Sigma-Aldrich, St. Louis, MO, USA) supplemented with 1% non-essential amino acids (Sigma-Aldrich, USA) and 10% FBS, at 37 °C, 5% CO_2_. For the experiments, HaCaT cells were seeded in 96-well plates at 10,000 cells/cm^2^ and, after 24 h, the medium was changed with the appropriate essential oil dilution as well as control (complete media). The essential oils were first diluted in DMSO at 100 mg/mL, and from this stock solution a range of serial dilutions were prepared in complete growth medium. The vehicle control consisting of the corresponding DMSO concentrations was also included.

Following 24 h of incubation with EOs, cell viability and cytotoxic effect were evaluated using XTT (MedChemExpress, Monmouth Junction, NJ, USA) and LDH (Sigma-Aldrich, St. Louis, MO, USA) assays according to the manufacturers’ instructions. The absorbance values for the XTT assay were measured at 450/650 nm using a TECAN microplate reader. The results were expressed as percentage of control (untreated cells).

For the level of lactate dehydrogenase (LDH), in addition to the EO samples and the negative control (complete culture medium), a positive control was also included by adding Tergitol to the cells, as provided in the kit. The absorbance values were measured at 500 nm, and the cytotoxicity was calculated as follows:

$$\frac{\left({OD}_{s}-{OD}_{-}\right)}{\left({OD}_{+}-{OD}_{-}\right)}\times 100$$

where OD is the optical density, and *s*, +, and − refer to the sample, positive control and negative control respectively.

##### Statistical Analysis

For the in vitro testing, data were expressed as mean values ± standard deviations (SD) from three independent experiments, with at least triplicates per condition, and the differences were calculated via the two-way ANOVA test using the GraphPad Prism 9 software. Statistical significance was considered at *p* < 0.05.

#### 3.3.4. Skin Compatibility Assessment by In Vivo Testing

The dermatological compatibility of the thuja and myrtle EOs was evaluated by the company Eurofins Evic Product Testing Romania SRL, using standardized epicutaneous patch testing under controlled conditions.

The patch test was conducted under controlled dermatological conditions following established guidelines for human skin compatibility assessment [50,51,52,53,54,55].

The study design adhered to recognized methodologies for single-application closed patch testing and irritation scoring frameworks, ensuring high reproducibility and comparability with published skin compatibility studies. Test products (Samples 1 to 5—diluted at 0.05% in ethanol) were applied in a standardized amount (20 µL) onto the upper back of the participants using occlusive Finn Chamber Standard^®^ patches (Smart Practice, 3400 E. McDowell Rd. Phoenix, AZ, SUA), with a contact time of 24 h. In parallel, a negative control (distilled water) and a positive control (1% sodium lauryl sulfate) were applied under the same testing conditions.

Skin reactions were evaluated before patch application, at 15–30 min after patch removal, and at 24 ± 2, 48 ± 4, and 72 ± 4 h after patch removal by a trained technician using validated irritation scoring scales (erythema, edema, dryness, and other visible signs of irritation).

For each tested subject and each experimental time point, an individual daily irritation score (IDIS) was calculated as the sum of the scores obtained for the clinical signs observed on the experimental area for the tested thuja and myrtle EOs, as well as for the negative and positive controls. For the panel and for each experimental time point, a mean daily irritation score (MDIS) was calculated according to the following formula:

$$MDIS=\;\frac{\sum_{i=1}^{n}\left(IDIS\right)\;}{n}\;$$

where *n* = number of valid cases.

The mean irritation index (MII) for tested thuja and myrtle EOs and controls was calculated according to the following formula:

$$MII=\;\frac{\sum_{i=1}^{m}\left(MDIS\right)\;}{m}\;$$

where *m* = number of experimental time points.

## 4. Conclusions

This study assessed the antimicrobial activity and cytotoxic activity of thuja and myrtle EOs. The best extraction yield for thuja EOs was obtained for dried cones. The main components of thuja EOs varied depending on the material type (leaves or cones) and moisture (fresh or dry) but included α-pinene, β-thujene, α-terpinolene, caryophyllene, cis-thujopsene, and cedrol for all EOs, while myrtle EO predominantly contained α-pinene, eucalyptol, α-terpineol, myrtenyl acetate, α-terpinyl acetate, and geranyl acetate. The antibacterial activity results revealed that this varied depending on the type of microbial strain tested and the chemical composition. Myrtle EO, which has a high concentration of oxygenated monoterpenes, displayed broad-spectrum activity by efficiently inhibiting the growth and adherence of both Gram-positive (*S. aureus*, *S. epidermidis*) and Gram-negative (*E. coli*) bacteria, as well as yeasts (*C. albicans*, *C. parapsilosis*). Thuja EOs showed selective activity against yeasts (*C. parapsilosis*) and Gram-positive bacteria (*S. aureus*). The EO extracted from dry thuja leaves was the most active among the thuja EO variations. The results revealed that the chemical profile and synergistic effects of the EOs’ constituent compounds influenced their antibacterial activity. In conclusion, all EOs, particularly myrtle, can be used as potential natural antimicrobial agents for preventing infections or biofilm formation on tissues and medical devices or for cosmetic application as natural preservatives.

Our in vitro results on human keratinocytes showed that all tested essential oils, at concentrations below 125 µg/mL, did not impair cell proliferation, thus supporting their use as a natural alternative to antibiotics for cutaneous applications, with no cytotoxic effects.

The patch test results demonstrated that, under the specified experimental conditions, the evaluated EOs (diluted to 0.05% in ethanol) exhibited good skin compatibility, which supports their suitability for potential topical applications.

## Figures and Tables

**Table 1:** Extraction yield for thuja and myrtle EOs.

EO Sample	Relative Density at 20 °C (g/mL)	Average Yield, g EO/100 g Vegetable Material
1-thuja fresh leaves	0.913 ± 0.003	0.210 ± 0.002
2-thuja dry leaves	0.942 ± 0.005	0.330 ± 0.006
3-thuja fresh cones	0.967 ± 0.004	0.510 ± 0.005
4-thuja dry cones	0.980 ± 0.003	1.630 ± 0.002
5-myrtle	0.894 ± 0.002	0.700 ± 0.001

The data are represented as mean ± SD (*n* = 3 for each analysis).

**Table 2:** Main components of myrtle oil (Sample 5) identified by GC-MS.

Peak	Compound	MF *	Mw * (g/mol)	RI *	t_R_ (min)	Area (%)
Monoterpene hydrocarbons						
1	**α-Pinene**	C_10_H_16_	136.125	937	8.731	**20.98**
2	*p*-Cymene	C_10_H_14_	134.11	1025	12.62	1.57
Total Monoterpene hydrocarbons						22.55
Oxygenated monoterpenes						
3	**Eucalyptol**	C_10_H_18_O	154.136	1032	12.973	**41.86**
4	Linalool	C_10_H_18_O	154.136	1099	16.168	4.95
5	**α-Terpineol**	C_10_H_18_O	154.136	1189	20.36	**7.7**
6	Myrtenol	C_10_H_16_O	152.12	1213	20.597	1.91
7	Geraniol	C_10_H_18_O	154.136	1255	23.34	1.78
Total Oxygenated monoterpenes						58.2
Monoterpenoid esters						
8	Linalyl acetate	C_12_H_20_O_2_	196.146	1257	23.434	2.43
9	**Myrtenyl acetate**	C_12_H_18_O_2_	194.131	1327	26.48	**4.72**
10	**α-Terpinyl acetate**	C_12_H_20_O_2_	196.146	1350	27.532	**6.99**
11	Geranyl acetate	C_12_H_20_O_2_	196.146	1382	29.047	3.03
Total Monoterpenoid esters						17.17
Total % identified						97.92

MF: molecular formula; Mw: molecular weight; RI: retention index; t_R_: retention time; Area (%): Percentage of each compound. * Theoretical values corresponding to the assigned compound.

**Table 3:** Main components of thuja EOs (Samples 1–4) identified by GC-MS.

Peak	Compound	MF *	Mw * (g/mol)		RI *	t_R_ (min)	Area (%)			
							Fresh		Dry	
							Leaves (Sample 1)	Cones (Sample 3)	Cones (Sample 4)	Leaves (Sample 2)
Monoterpene hydrocarbons										
1	**α-Thujene**	C_10_H_16_	136.13		929	8.46	-	0.92	**3.89**	1.99
2	**α-Pinene**	C_10_H_16_	136.13		937	8.69–8.80	**65.78**	**29.12**	**33.65**	**30.02**
3	Camphene	C_10_H_16_	136.13		952	9.28	-	0.34	0.54	0.38
4	**β-Thujene**	C_10_H_16_	136.13		974	10.35	**3.47**	**4.66**	**12.09**	**6.54**
5	β-Pinene	C_10_H_16_	136.13		979	10.45	1.7	2	2.39	1.62
6	**β-Myrcene**	C_10_H_16_	136.13		991	11.17	1.56	**3.07**	**4**	**3.2**
7	α-Phellandrene	C_10_H_16_	136.13		1005	11.68	-	0.38	-	0.7
8	**3-carene**	C_10_H_16_	136.13		1011	11.94	**4.08**	**6.62**	1.69	**3**
9	α-Terpinene	C_10_H_16_	136.13		1017	12.24	-	0.36	2.78	0.58
10	*p*-Cymene	C_10_H_14_	134.11		1025	12.6	2.25	1.33	0.47	1.41
11	Limonene	C_10_H_16_	136.13		1031	12.78	3.11	2.91	2.92	3.12
Oxygenated monoterpenes										
12	Eucalyptol	C_10_H_18_O	154.14		1032	12.88	1.51	0.36	-	-
Monoterpene hydrocarbons										
13	**γ-Terpinene**	C_10_H_16_	136.13		1060	14.19	-	0.67	**4.33**	1
14	**α-Terpinolene**	C_10_H_16_	136.13		1088	15.56	-	**3.1**	**4.23**	**2.43**
Total Monoterpene hydrocarbons							81.95	55.48	72.98	55.99
Oxygenated monoterpenes										
15	**Terpinen-4-ol**	C_10_H_18_O	154.14		1177	19.70–19.74	-	1.18	**10.68**	2.92
16	α-Terpineol	C_10_H_18_O	154.14		1189	20.33	-	-	0.84	-
Total Oxygenated monoterpenes							1.51	1.54	11.52	2.92
Monoterpenoid esters										
17	Borneol acetate	C_12_H_20_O_2_	196.15		1293	24.71	0.86	2.38	1.81	1.36
18	α-Terpinyl acetate	C_12_H_20_O_2_	196.15		1350	27.51	0.89	2.52	1.02	1.59
Total Monoterpenoid esters							1.75	4.90	2.83	2.95
Sesquiterpene hydrocarbons										
19	Cedrene	C_15_H_24_	204.19		1410	30.12	0.68	1.75	0.49	1.51
20	**Caryophyllene**	C_15_H_24_	204.19		1419	30.44–30.45	**2.9**	**5.72**	1.74	**5.63**
21	***cis*-Thujopsene**	C_15_H_24_	204.19		1429	30.9	**4.07**	**5.58**	1.48	**3.82**
22	**Humulene**	C_15_H_24_	204.19		1454	31.84	1.74	**3.45**	0.96	**3.98**
23	Germacrene D	C_15_H_24_	204.19		1481	32.98	-	1.65	0.71	2.29
24	Cuparene	C_15_H_22_	202.17		1505	33.97	-	0.89	-	0.54
Total Sesquiterpene hydrocarbons							9.39	19.04	5.38	17.77
Oxygenated sesquiterpenes										
25	Khusian-2-ol	C_15_H_26_O	222.2		1599	37.21	-	1.32	0.47	1.92
26	**Cedrol**	C_15_H_26_O	222.2		1599	37.65–37.72	**5.40**	**10.95**	**5.54**	**16.52**
27	α-Acorenol	C_15_H_26_O	222.2		1630	38.76	-	1.32	-	0.67
Total Oxygenated sesquiterpenes							5.40	13.59	6.01	19.11
				Total % identified			100	94.55	98.72	98.74

MF: molecular formula; Mw: molecular weight; RI: retention index; t_R_: retention time; Area (%): percentage of each compound. * Theoretical values corresponding to the assigned compound.

**Table 4:** Quantitative and qualitative evaluation of antimicrobial activity expressed by diameter of the inhibition zone (DIZ), minimum inhibitory concentration (MIC), minimum microbicidal concentration (MMC), and minimum inhibitory concentration of microbial adherence (MICMA). All DIZ values are expressed in mm, while MIC, MMC, and MICMA are expressed in µL/mL.

Sample	Determined Parameter	*S. aureus* ATCC 25923	*S. epidermidis* ATCC 12228	*P. aeruginosa* ATCC 27853	*E. coli* ATCC 25922	*C. albicans* ATCC 10231	*C. parapsilosis* ATCC 22019
**1-Thuja fresh leaves**	DIZ	8.5 ± 0.7	8.5 ± 0.7	5.0 ± 0.0	5.5 ± 0.7	9.0 ± 0.0	5.5 ± 0.7
	MIC	1.953	125	>125	125	31.25	15.625
	MMC	1.953	125	>125	125	125	62.5
	MICMA	7.813	**62.5**	**62.5**	**62.5**	62.5	**7.8125**
**2-Thuja dry leaves**	DIZ	12.5 ± 0.7	11 ± 1	5.5 ± 0.7	6.0 ± 0.0	10.0 ± 0.0	11.5 ± 0.7
	MIC	15.625	62.5	62.5	31.25	7.8125	7.8125
	MMC	31.25	31.25	62.5	62.5	31.25	125
	MICMA	**3.906**	62.5	62.5	62.5	15.625	**3.906**
**3-Thuja fresh cones**	DIZ	5.5 ± 0.7	9.5 ± 0.7	6.5 ± 0.7	10.5 ± 0.7	9.5 ± 0.7	6.5 ± 0.7
	MIC	7.813	31.25	125	15.625	15.625	7.8125
	MMC	7.813	62.5	125	15.625	62.5	62.5
	MICMA	7.813	31.25	125	**7.813**	15.625	7.8125
**4-Thuja dry cones**	DIZ	10.5 ± 0.7	9 ± 2	7.0 ± 0.0	0.0 ± 0.0	9.0 ± 0.0	8.5 ± 0.7
	MIC	>125	62.5	62.5	62.5	31.25	7.8125
	MMC	>125	>125	62.5	62.5	>125	62.5
	MICMA	>125	62.5	62.5	62.5	62.5	7.8125
**5-Myrtle**	DIZ	18 ± 3	11.5 ± 0.7	8.5 ± 0.7	16 ± 3	18 ± 4	17 ± 1
	MIC	3.096	7.813	7.813	3.096	7.813	7.813
	MMC	15.625	7.8125	7.813	15.625	125	62.5
	MICMA	7.813	7.813	7.813	3.096	7.813	7.813
**DMSO (negative control)**	DIZ	0.0 ± 0.0	0.0 ± 0.0	0.0 ± 0.0	0.0 ± 0.0	0.0 ± 0.0	0.0 ± 0.0
	MIC	250	>250	125	125	125	62.5
	MMC	>250	>250	250	>250	250	125
	MICMA	>250	>250	125	125	125	62.5
**Gentamicin**	MIC (µg/mL)	1.09	0.55	4.38	17.5	-	-
	MMC (µg/mL)	17.5	4.38	8.75	70	-	-
	MICMA (µg/mL)	17.5	1.09	4.38	17.5	-	-
**Ketoconazole**	MIC (µg/mL)	-	-	-	-	8.75	1.09
	MMC (µg/mL)	-	-	-	-	70	2.19
	MICMA (µg/mL)	-	-	-	-	17.5	1.09

## Data Availability

Data cannot be made publicly available due to privacy and ethical restrictions (human subject testing). The summary statistics supporting the reported results are provided within the article. Anonymized data may be available from the corresponding author upon reasonable request and with institutional ethics approval.

## Supplementary Materials

The following supporting information can be downloaded at: https://www.mdpi.com/article/10.3390/molecules31071225/s1, Figure S1. GC-MS total ion chromatogram for myrtle oil (Sample 5); Figure S2. GC-MS total ion chromatogram for thuja oil extracted from fresh leaves (Sample 1); Figure S3. GC-MS total ion chromatogram for thuja oil extracted from dry leaves (Sample 2); Figure S4. GC-MS total ion chromatogram for thuja oil extracted from fresh cones (Sample 3); Figure S5. GC-MS total ion chromatogram for thuja oil extracted from dry cones (Sample 4).
