# Supplementary material for: Chemical Characterization, Evaluation of Antimicrobial Potential, and Cytotoxic Activity of Thuja occidentalis L. and Myrtus communis L. Essential Oils for Topical Applications
Source: Molecules. 2026 Apr 7;31(7):1225. doi: 10.3390/molecules31071225 (PMC13074968; doi:10.3390/molecules31071225)
Supplement: Supplementary file 1 [file molecules-31-01225-s001.zip › molecules-4237248-supplementary.pdf]

Supplementary Information

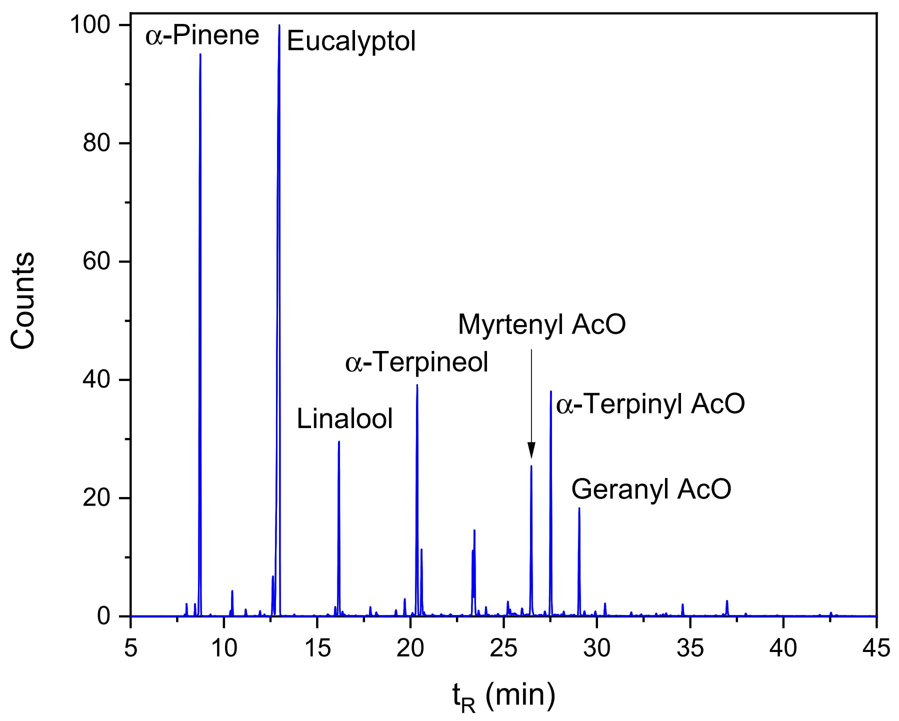

Fig. S1. GC-MS Total ion chromatogram for myrtle oil (Sample 5).

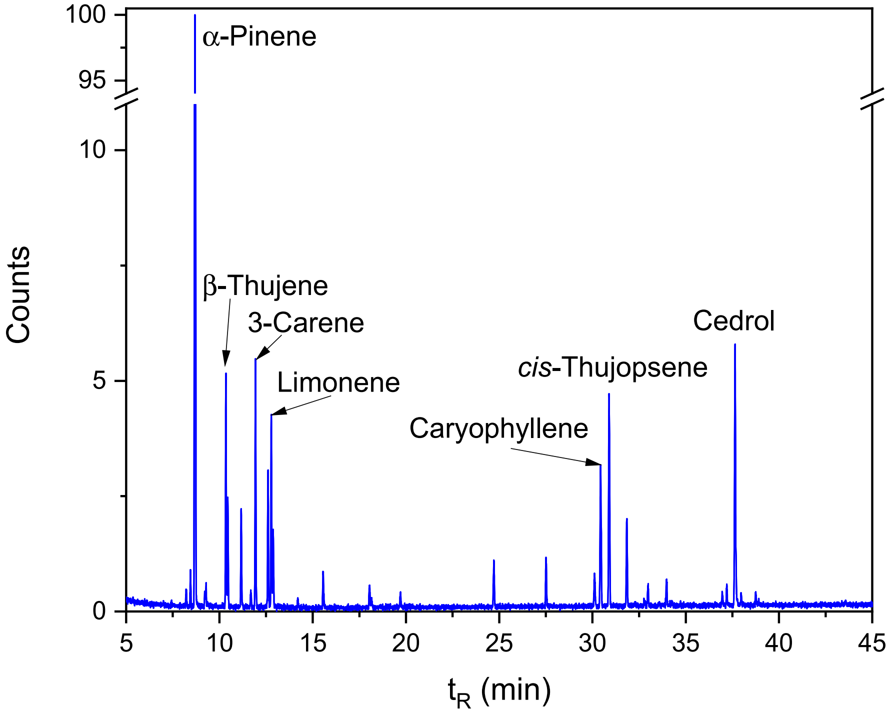

Fig. S2. GC-MS Total ion chromatogram for thuja oil extracted from fresh leaves (Sample 1).

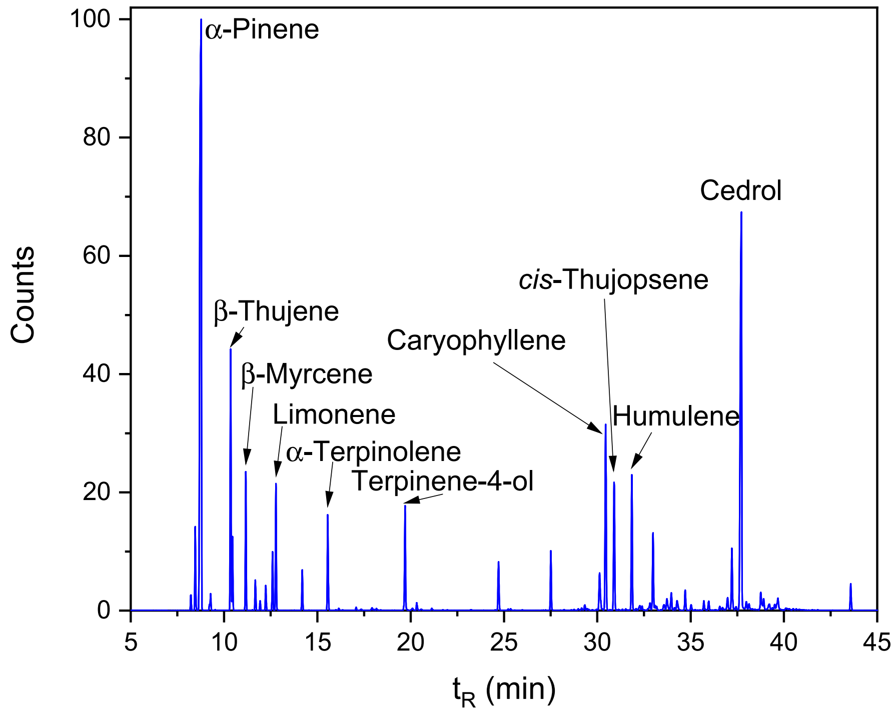

Fig. S3. GC-MS Total ion chromatogram for thuja oil extracted from dry leaves (Sample 2).

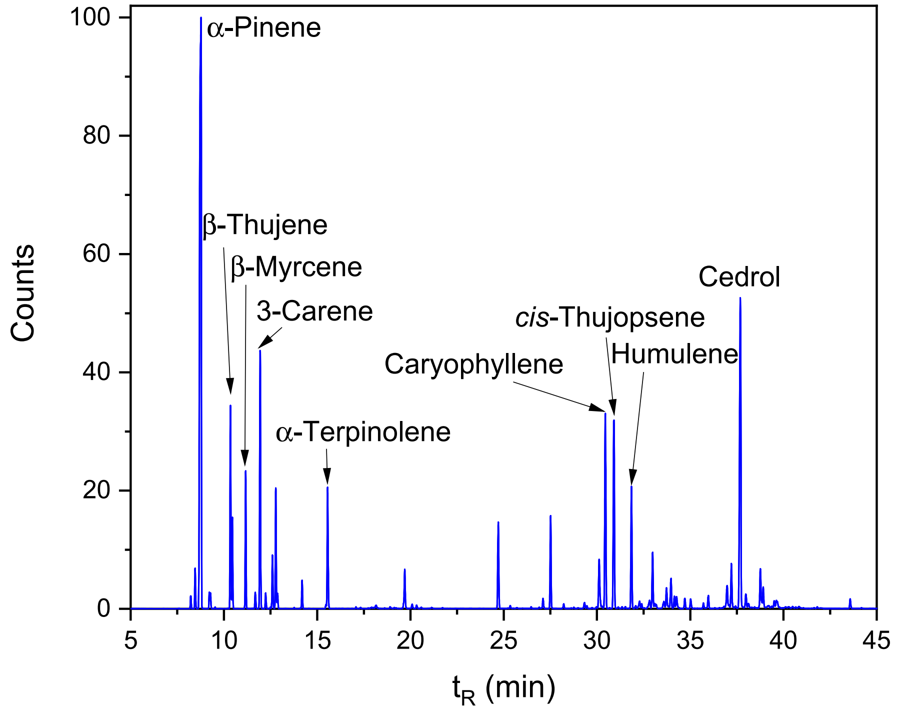

Fig. S4. GC-MS Total ion chromatogram for thuja oil extracted from fresh cones (Sample 3).

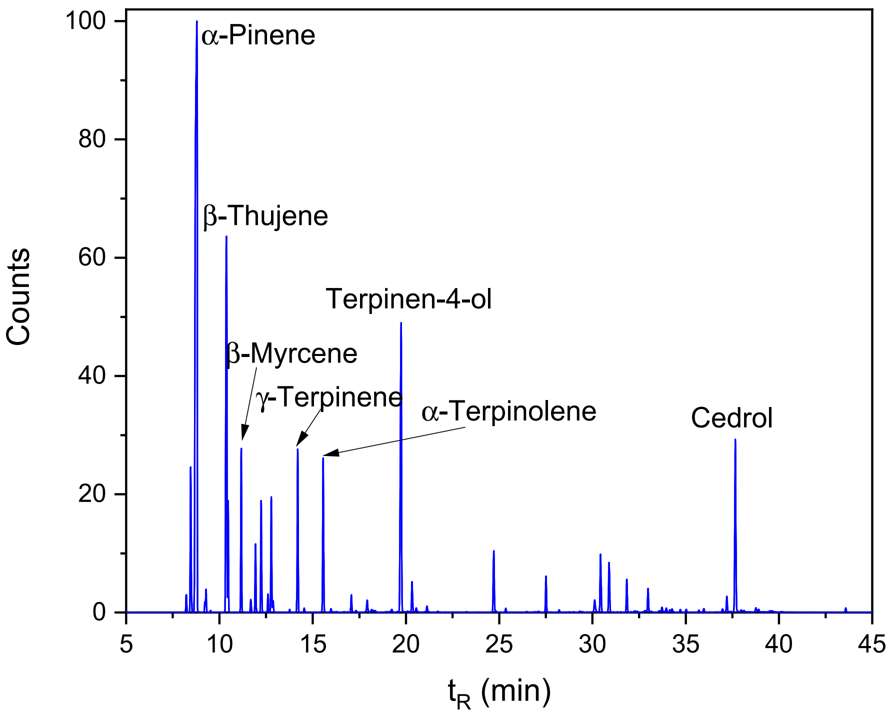

Fig. S5. GC-MS Total ion chromatogram for thuja oil extracted from dry cones (Sample 4).
